# Supplementary material for: Genetic designs for stochastic and probabilistic biocomputing
Source: Phys Rev E. Author manuscript; Available in PMC 2026 Mar 21. (PMC7618911; doi:10.1103/PhysRevE.111.054412)
Supplement: Supplementary [file EMS212933-supplement-Supplementary.pdf]

# Supplementary Information

# Genetic designs for stochastic and probabilistic biocomputing

## S1 Measurements and experimental setups for random pulse computing

### S1.1 In silico pulse counting

In computational simulations, pulses are identified according to a z-score algorithm. In this algorithm, given a time series of protein expression/abundance, a pulse is identified as a region for which protein expression/abundance rises a threshold number of standard deviations above a simple moving average (mean) of protein expression. Both the threshold and the window size of the moving average can be adapted to reflect an expected maximum pulse amplitude and pulse rate — for example, the algorithm is most effective if any particular window includes only one occurrence of a pulse.

### S1.2 Experimental pulse counting

Although we have not conducted any experimental validation for the circuit designs presented here, we can outline here an idea of an experimental setup for measuring the outputs of our proposed genetic RPC circuits.

Basically, although it might be experimentally difficult to explicitly measure the frequency of pulses in a single cell (although it could be possible using sophisticated optogenetic setups, for example), a approximation of the pulse frequency can be made by measuring the state of a whole population of cells at once. At any given time, some proportion of population will be emitting a pulse, and this proportion can be used to estimate the frequency of the pulses, given the size of the population.

In the example of using a fluorescent protein as an output, this proportion could be estimated by passing the population through a cytometer. This might not be necessary, however, if the proportion could be approximated by a population level fluorescence signal, in which case a plate reader or similar could be used.

Figure S3 outlines this kind of experimental setup. At the bottom of Figure S3, we show the results of simulations of entire populations of pulse repeater circuits. We show a timecourse of the population average abundance of the output molecule, under different input pulse frequencies, and we see that output levels are roughly proportional to input pulse frequency.

### S1.3 Sensitivity of pulse characteristics

In practice, the width and amplitude of pulses in a pulse train will fluctuate. One concern is that there is some probability that small amplitude pulses will occur that will not be detected or propagated

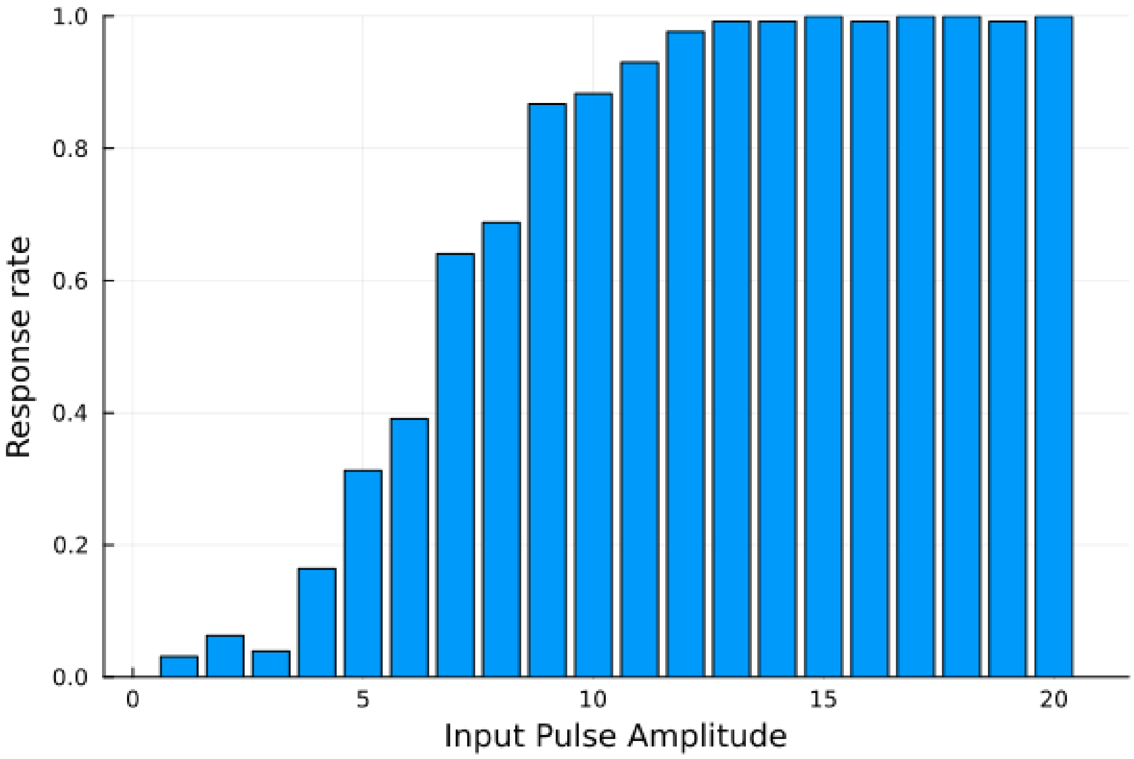


Figure S1: **Response rate of a pulse repeater as a function of the amplitude of the input pulse, in proteins.** The expected response is a pulse at the IFFL output as defined in section S1.1. Lower input pulse amplitudes show lower response rates.

properly, and this is an source of error in for the random pulse computing designs presented.

However, our simulations leave us with the impression that the rates of these kinds errors are quite low for a broad range of amplitudes. An example is shown in Figure S1.

Another advantage of our RPC designs with respect to these errors is that small amplitude input pulses, if they are detected and propagated, do not tend to be propagated as small amplitude output pulses. The example shown in Figure S2 (top), illustrates a lack of correlation between input and output pulse amplitude (beyond a certain threshold). In Figure S2 (bottom) we see that a simulated pulse repeater actually produces a tighter distribution of output pulse amplitudes than the input pulse amplitude distribution. The suggestion is that the pulse repeater is “cleaning up” the input signal.

Based on these results for pulse amplitude, we would not expect a blow up of noise across large RPC genetic circuits, which is a useful property. In fact, we should expect noise to be dampened with increasing depth of computational paths in a RPC genetic circuit.

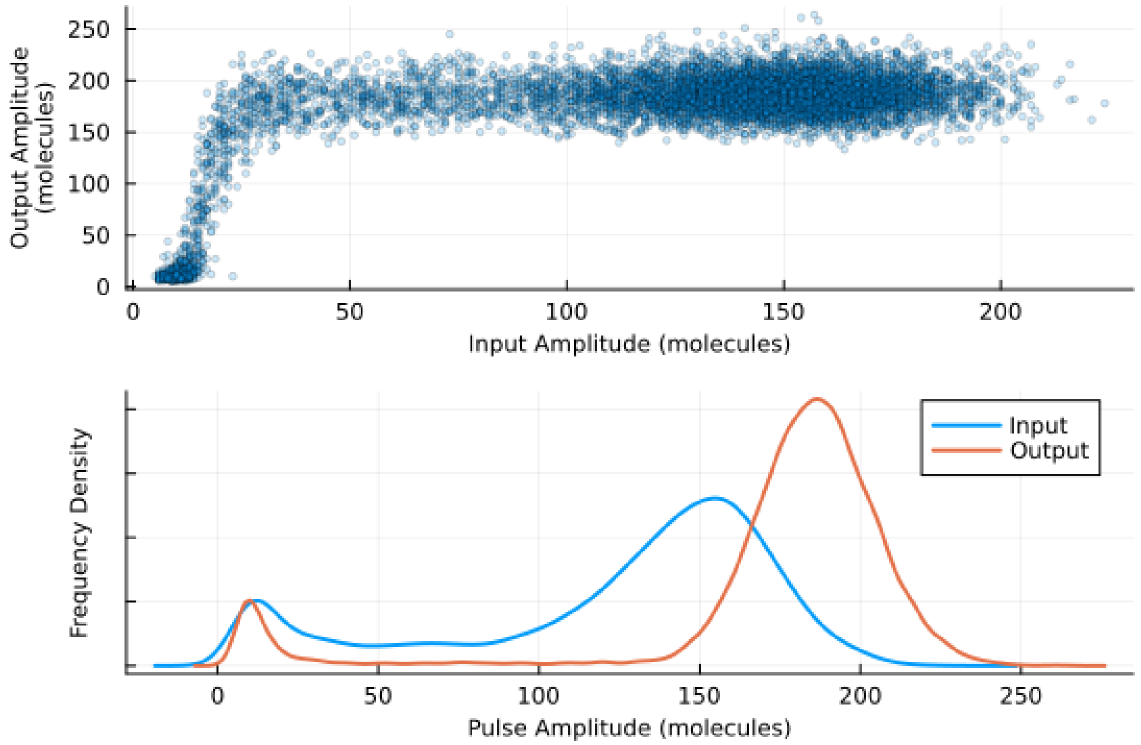


Figure S2: **Results of stochastic simulations of a pulse repeater.** The amplitude of the input pulse is varied and the amplitude of the output pulse produced by the repeater is measured. In the top panel, each dot represents the input and output amplitudes from one simulation. We can see that small amplitude inputs produce an highly variable output amplitude. However, once input amplitude reaches a threshold, the variance of output amplitude drops dramatically while the mean amplitude increases, even if the input amplitude shows high variance. The dynamics of the pulse repeater reduce the coefficient of variation in pulse amplitudes. In the bottom panel, the same simulation results are plotted as two probability densities, one for the input amplitude (blue) and one for the output amplitude (orange). We can see more clearly the lower variance and higher mean of the output amplitude relative to the input amplitude.

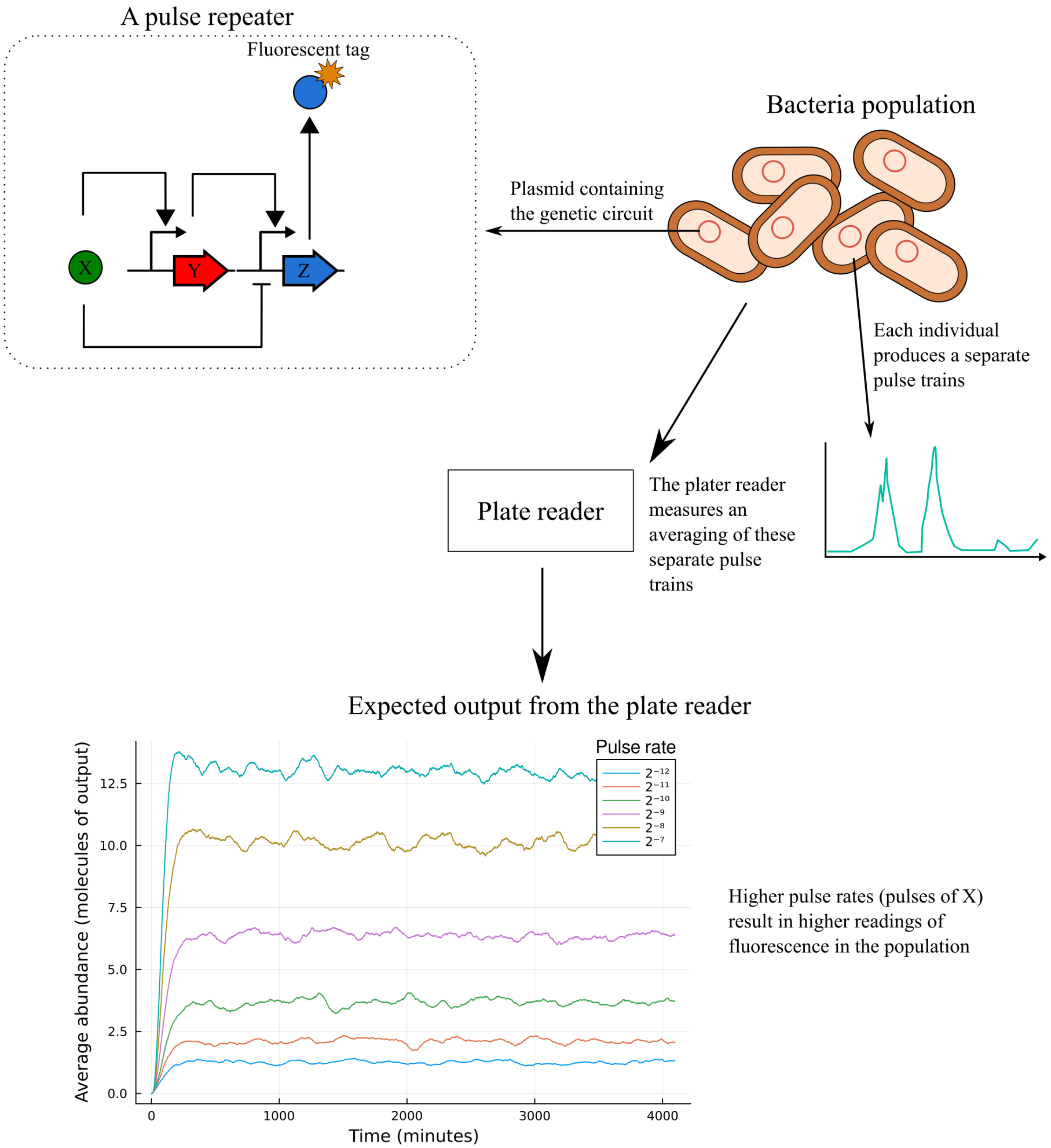


Figure S3: **A sketched outline of an experiment that could be used to estimate the output pulse frequency of a genetic RPC circuit.** The circuit, in this example a pulse repeater, in hosted by a population of bacteria. Each bacteria produces its own output pulses of Z (which could be a fluorescent protein or tag). Although it may be difficult to directly observe the frequencies of individual outputs, measuring the signal across the whole population in a device such as plate reader could give an estimate of frequency of pulses in individual bacteria. In our simulations (example shown in figure), the population's average expression of Z was roughly proportional to population's average pulse frequency of Z.

# S2 Experimental implementation and feasibility of p-bits

While this work focuses on computational models and simulations, here we outline how these circuits could be validated in living cells. Specifically, we have performed additional *in silico* experiments (S2.1 and S2.2 below) illustrating how transcription factor (TF) levels correlate with the p-bit state, and we propose an experimental design (S2.3 below) demonstrating how the two-p-bits NOT gate might be constructed and monitored in a laboratory setting.

## S2.1 Correlating p-bit state with TF protein levels

Figures S4 and S6 demonstrate the direct correlation between a gene's 0/1 p-bit state and the concentration of its encoded TF proteins. Specifically:

- **Figure S4 (NOT Gate).** We show that when Gene 1 (input) is in the 1 (p-bit=1) state, it produces a high number of TF proteins, whereas Gene 2 (output) is strongly repressed and has correspondingly fewer TF molecules, indicating p-bit=0. Conversely, switching occurs when Gene 2 is 1 and Gene 1 is 0.
- **Figure S6 (AND Gate).** A similar relationship holds for the three-p-bits AND gate: each gene's p-bit state (0/1) maps to a distinct TF protein count, enabling the logic function. For instance, when Genes 1 and 2 (inputs) are both 1, Gene 3 (output) is also driven to 1, reflecting a high TF count in all three. When either or both inputs are 0, the TF count for Gene 3 remains low.

In a laboratory setting, these correlations can be measured by tagging each TF with a fluorescent marker—e.g., GFP or RFP—and monitoring the resultant fluorescence. High fluorescence signals correspond to transcriptionally active states (p-bit=1), whereas low or baseline signals correspond to transcriptionally repressed states (p-bit=0).

## S2.2 Dynamics of p-bits

Figures S5 and S7 illustrate how each p-bit randomly toggles between 0 and 1 states, leading to fluctuations in the corresponding TF protein counts:

- **Figure S5 (NOT Gate).** We track TF copy numbers for each gene, showing periods when Gene 1 remains high (Gene 2 low) and transitions to the opposite state. These switching events provide insight into the probabilistic nature of gene expression.
- **Figure S7 (AND Gate).** The three-gene system exhibits more complex, yet still clearly discernible, switching patterns. Depending on the interplay among Genes 1, 2, and 3, the system explores different stable configurations with varying TF protein levels.

Experimentally, fluorescent reporters provide a powerful means to capture these stochastic transitions. A plate reader or frequent sampling with a flow cytometer can measure the distribution of p-bit states across a population over time, thereby revealing the system's switching rates and probabilities.

### S2.3 Illustrative design for the NOT gate

To exemplify a potential experimental approach, Figure S8 shows a plasmid-based implementation of our two-p-bits NOT gate. Each gene is fused to a distinct fluorescent protein—green (GFP) and red (RFP)—so that the p-bit states are directly observable. Growing cells carrying this plasmid allows continuous monitoring of the GFP/RFP output using a plate reader for population-level metrics, or single-cell flow cytometry to capture stochastic variability among individual cells. By correlating fluorescent intensities with each gene's activity, one can unambiguously determine the p-bit states. This same approach can be readily extended to the three-gene AND gate or more complex p-bit circuits. Taken together, these design principles and experiments provide a concrete roadmap for bringing p-bit-based genetic circuits from computational models into real-world biological implementations.

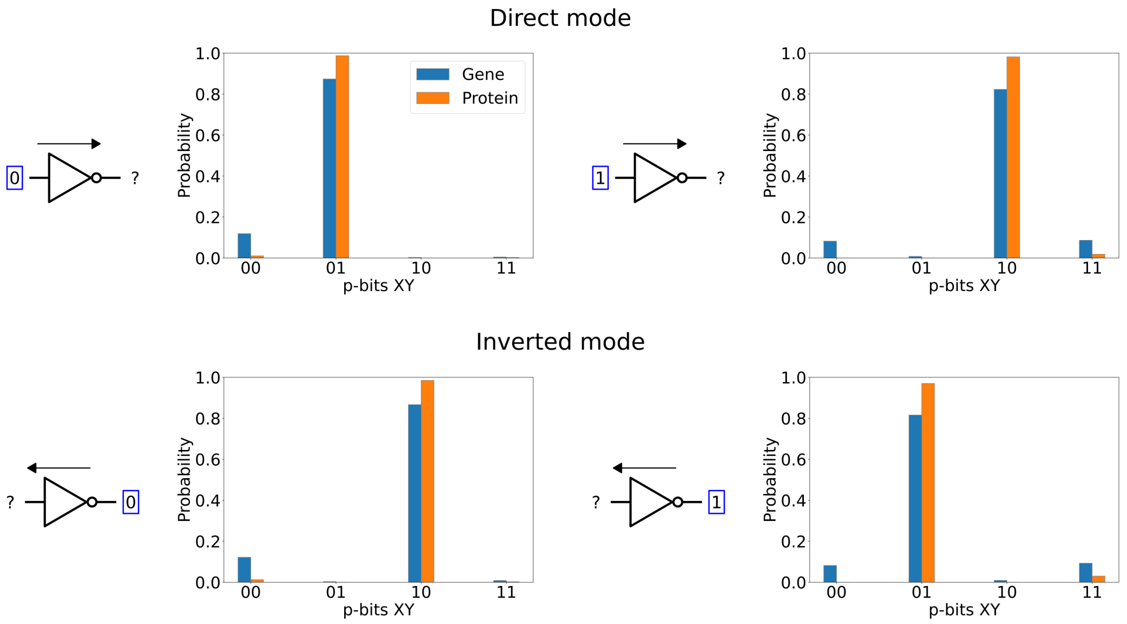


Figure S4: **Correlation between p-bit state and number of proteins in the two-p-bits NOT gate.** This figure shows the operation of the invertible p-bit NOT gate and illustrates the correlation between the state of the p-bits and the number of transcription factor (TF) proteins.

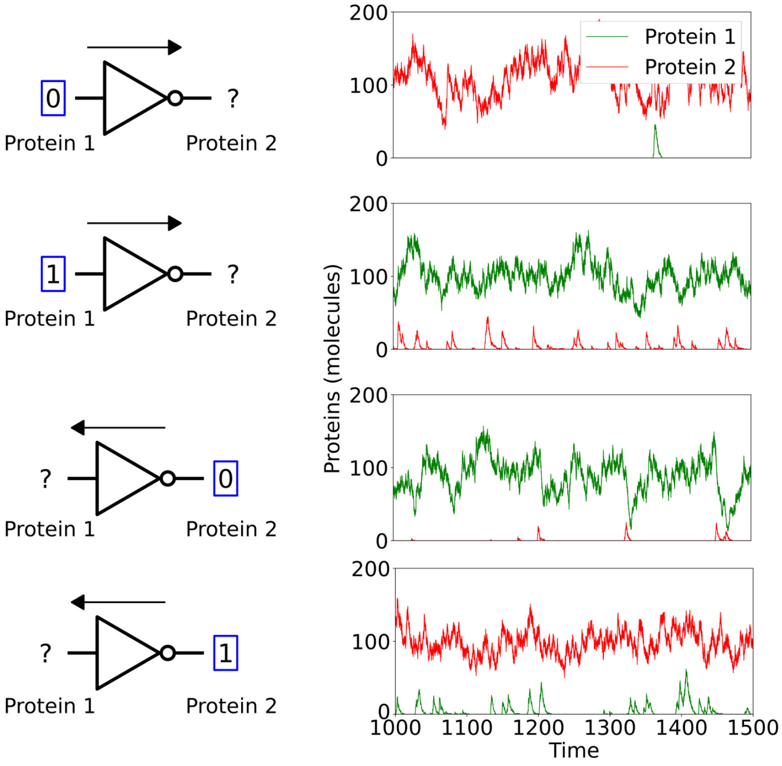


Figure S5: **Time evolution of TF proteins in the two-p-bits NOT gate.** This figure depicts the time evolution of the number of TF proteins for the NOT gate. The dynamic changes in TF concentrations over time are shown, illustrating how fluorescence proteins would appear. These fluorescence proteins can be measured using a plate reader or flow cytometer, providing insight into the p-bit state changes.

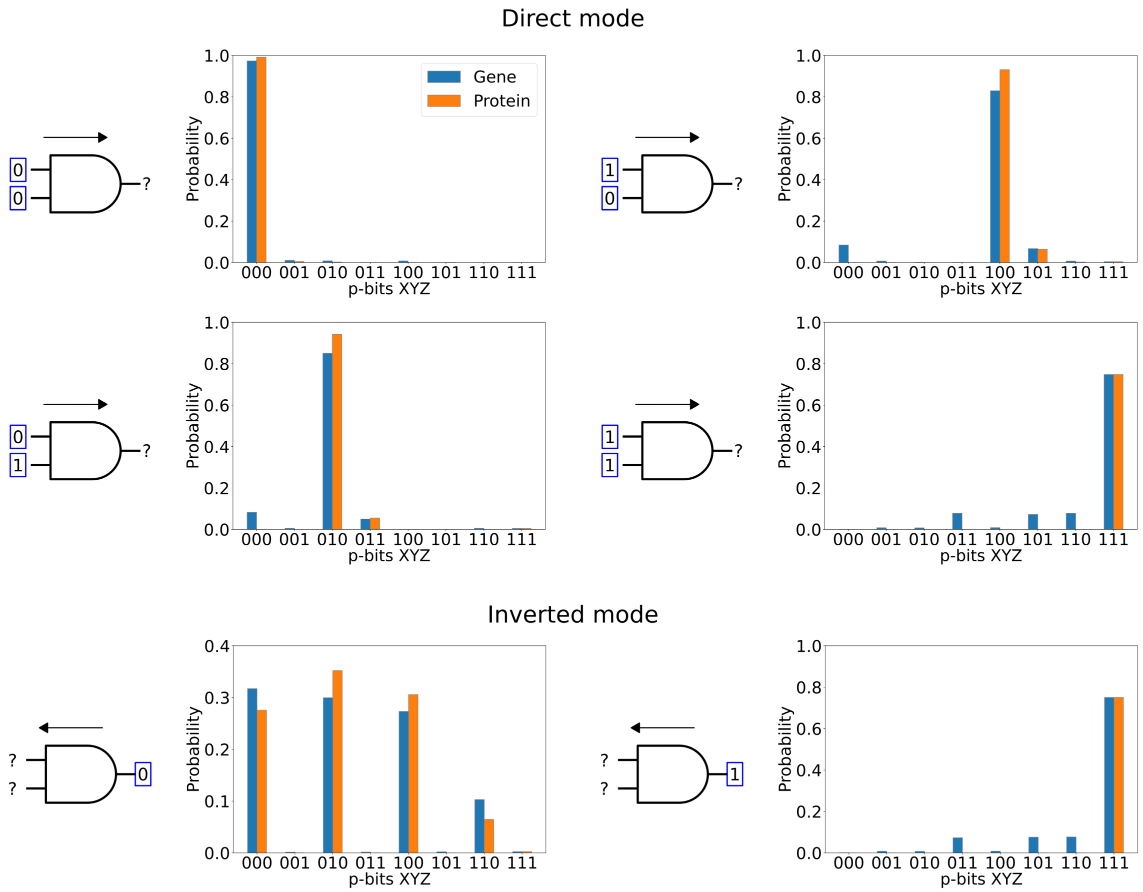


Figure S6: **Correlation between p-bit state and number of proteins in the three-p-bits AND gate.** This figure shows the operation of the invertible p-bit AND gate and illustrates the correlation between the state of the p-bits and the number of transcription factor (TF) proteins.

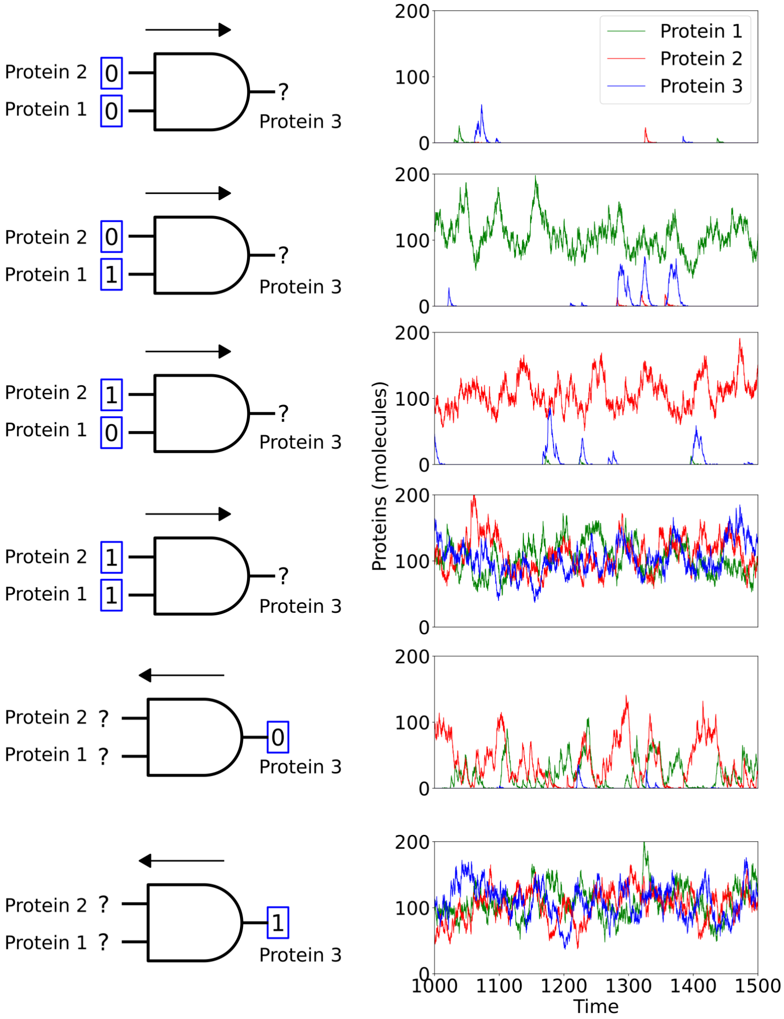


Figure S7: **Time evolution of TF proteins in the three-p-bits AND gate.** This figure depicts the time evolution of the number of TF proteins for the AND gate. The dynamic changes in TF concentrations over time are shown, illustrating how fluorescence proteins would appear. These fluorescence proteins can be measured using a plate reader or flow cytometer, providing insight into the p-bit state changes.

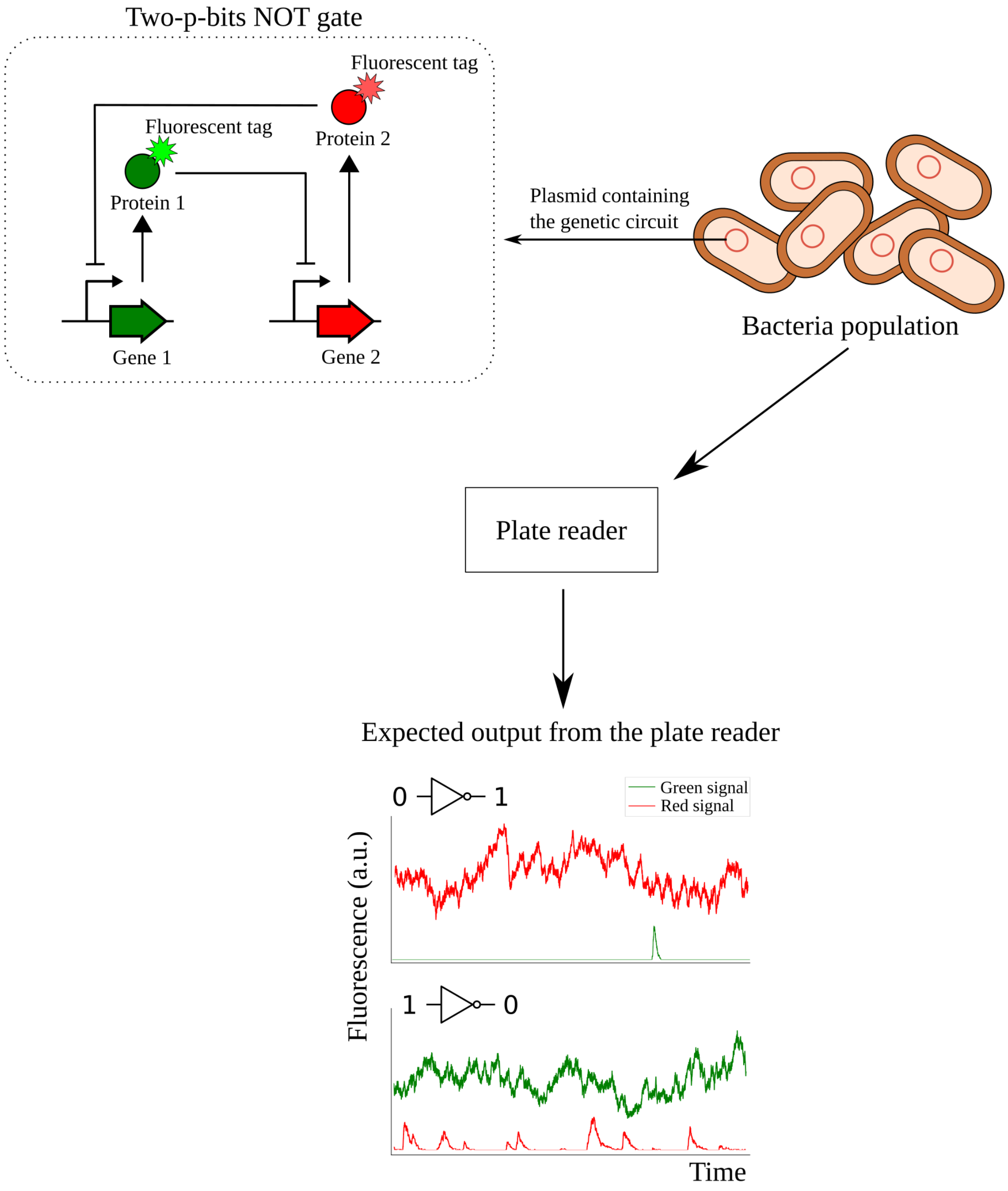


Figure S8: **Experimental schematic for measuring the two-p-bits NOT gate.** The diagram illustrates a plasmid design containing the two-gene NOT gate circuit. Gene 1 is tagged with a green fluorescent protein (GFP) reporter, and Gene 2 is tagged with a red fluorescent protein (RFP) reporter. Cells carrying this plasmid are grown under standard culture conditions and sampled at regular intervals. The GFP and RFP signals are measured either by plate reader (for population-level fluorescence) or by flow cytometry (to capture single-cell fluorescence distributions). Variations in GFP/RFP intensity reveal the probabilistic switching (p-bit $= 0$ or $1$) of Gene 1 and Gene 2, allowing direct experimental validation of the invertible NOT gate design.
